# A new nomogram including total cerebral small vessel disease burden for individualized prediction of early-onset depression in patients with acute ischemic stroke

**DOI:** 10.3389/fnagi.2022.922530

**Published:** 2022-09-27

**Authors:** Lihua Zhou, Licong Chen, Linqing Ma, Shanshan Diao, Yiren Qin, Qi Fang, Tan Li

**Affiliations:** ^1^Department of Neurology, The People's Hospital of Suzhou New District, Suzhou, China; ^2^Department of Neurology, The First Affiliated Hospital of Soochow University, Suzhou, China

**Keywords:** cerebral small vessel disease burden, early-onset depression, acute ischemic stroke, nomogram, prediction

## Abstract

**Objectives:**

The present study was designed to evaluate the effects of total cerebral small vessel disease (CSVD) on early-onset depression after acute ischemic stroke (AIS), and to develop a new nomogram including total CSVD burden to predict early-onset post-stroke depression (PSD).

**Methods:**

We continuously enrolled patients with AIS who were hospitalized at the First Affiliated Hospital of Soochow University between October 2017 and June 2019. All patients were assessed for depressive symptoms using the 17-item Hamilton Depression Scale (HAMD-17) at 14 ± 2 days after the onset of AIS. The diagnosis for depression was made according to the American Diagnostic and Statistical Manual of Mental Disorders Version 5 (DSM-5). The demographic and clinical data were collected including total CSVD burden. On the basis of a multivariate logistic model, the independent factors of early-onset PSD were identified and the predictive nomogram was generated. The performance of the nomogram was evaluated by Harrell's concordance index (C-index) and calibration plot.

**Results:**

A total of 346 patients were enrolled. When contrasted to a 0 score of total CSVD burden, the score ≥2 (moderate to severe total CSVD burden) was an independent risk factor for early-onset PSD. Besides, gender, cognitive impairments, baseline Barthel Index (BI), and plasma fibrinogen were independently associated with early-onset PSD. The nomogram based on all these five independent risk factors was developed and validated with an Area Under Curve (AUC) of 0.780. In addition, the calibration plot revealed an adequate fit of the nomogram in predicting the risk of early-onset depression in patients with AIS.

**Conclusions:**

Our study found the total CSVD burden score of 2–4 points was an independent risk factor of early-onset PSD. The proposed nomogram based on total CSVD burden, gender, cognitive impairments, baseline BI, and plasma fibrinogen concentration gave rise to a more accurate and more comprehensive prediction for early-onset PSD.

## Introduction

Post-stroke depression (PSD) is the most common emotional disorder after acute ischemic stroke (AIS) (Hackett and Pickles, 2014), which can increase the risk of stroke mortality (House et al., 2001; Williams et al., 2004), cause recurrence of stroke (Yuan et al., 2012) and lead to poor functional prognosis (Pohjasvaara et al., 2001; Ayerbe et al., 2014). About 31% of AIS patients may suffer from PSD during any period after stroke (Hackett and Pickles, 2014). Degree of physical disability, cognitive impairment, stroke severity, female gender and lack of social and family support are considered as the risk factors for PSD (Hackett and Anderson, 2005). Early-onset PSD refers to depression that occurs within 2 weeks after the onset of stroke (Altieri et al., 2012; He et al., 2017; Huang et al., 2018; Zhao et al., 2018). Compared to late-onset PSD, early-onset PSD possesses more depressive symptoms (Tateno et al., 2002) and may reduce patients' desire for drug and rehabilitation therapy (Willey et al., 2010). Thus, early detection and intervention of PSD are important to improve the prognosis of AIS patients (Gabaldón et al., 2007).

Cerebral small vessel disease (CSVD) refers to a group of pathological processes that affect the small arteries, arterioles, venules, and capillaries of the brain (Pantoni, 2010). CSVD may result in various clinical symptoms including cognitive disorders, dementia, balance disorders, gait disorders and emotional disorders (Wardlaw et al., 2013; van Agtmaal et al., 2017). Neuroimaging characteristics of CSVD include white matter hyperintensities (WMHs), silent lacunar infarction (sLI), enlarged perivascular spaces (EPVS), and cerebral microbleeds (CMBs) (Pantoni, 2010; Wardlaw et al., 2013). Nowadays, more and more existing evidence indicates that CSVD could be one of the crucial factors of PSD (Santos et al., 2009b). However, recent studies mostly focus on the relationship between PSD and a specific CSVD subtype. In fact, the coexistence of various subtypes of CSVD in one patient is common, thus the total CSVD burden may be a better indicator to reflect the severity of CSVD. Staals et al. developed a CSVD global burden rating scale including the WMHs, sLI, EPVS, and CMBs subtypes (Staals et al., 2014). The practicality and reliability of the rating scale have been verified in many studies (Staals et al., 2015; Lau et al., 2017; Jiang et al., 2019; Liu et al., 2019; Wu et al., 2020). However, the relationship between the total CSVD burden with PSD is poorly studied.

Nomogram, a graphical statistical instrument, has been generally used in medical decision-making. To date, there is no nomogram model to predict the risk of early-onset PSD. Therefore, the purposes of this study were to investigate the effects of CSVD on early-onset PSD and to build a nomogram as the prediction model of early-onset PSD. We assumed that total CSVD burden was a prominent risk factor for early-onset PSD, and the nomogram could be a promising prediction tool.

## Materials and methods

### Study subjects

The present study was a retrospective analysis with collected data from the neurological department of First Hospital Affiliated of Soochow University between October 2017 and June 2019. All patients suffered from AIS within 3 days. Patients with the following conditions were excluded: depressive disorders, anxiety disorders, or other mental illnesses diagnosed by the professional psychiatrists before the stroke onset; accompanied by severe systemic diseases; medical histories of other central nervous system diseases; previous histories of alcohol or drug abuse; disturbance of consciousness, severe language disorders, Mini-Mental State Examination (MMSE) scale lower than 17 points or other situations that resulted in failure to complete the mental psychological assessment; not able to conduct an MRI examination. Ethical approval for this study was obtained from the institutional review board of the First Affiliated Hospital of Soochow University (2022310). The data were anonymous, and the requirement for informed consent was therefore waived.

### Data collection and baseline evaluation

Demographic and clinical baseline data were collected, including gender, age, smoking and alcohol consumption, past disease history, education level, baseline NIHSS scale, baseline Barthel Index (BI), TOAST type, baseline blood pressure, baseline blood glucose and lipid levels, homocysteinemia and other relevant laboratory data. TOAST type was adjusted according to further examination results before discharge.

### Clinical assessment

17-item Hamilton Depression Scale (HAMD-17) was adopted at 14 ± 2 days after the onset of AIS to evaluate mental disorders of the patients. HAMD-17 points <7 means normal condition; mild depression for 7–17 points; moderate depression for 18–24 points; more than 24 points for severe depression (Zhu et al., 2016; He et al., 2017; Luan et al., 2020; Chi et al., 2021). The diagnosis was made according to the American Diagnostic and Statistical Manual of Mental Disorders Version 5 (DSM-5). MMSE scale was conducted to evaluate patients' cognitive function. Cognitive impairment was defined according to the MMSE score and education level referred to as standard. HAMD-17 and MMSE were conducted by two independent neuropsychological experts.

### The assessment of CSVD

The magnetic resonance imaging (MRI) sequences involved in the study included diffusion-weighted imaging (DWI), 3d-TOF-MRA, FLAIR, T2-weighted, T1-weighted, and susceptibility weighted imaging (SWI).

The total CSVD burden score consisted of four imaging markers including WMHs, EPVS, CMBs, and sLI. When WMHs were graded according to Fazekas' scale, one point was awarded as either irregular periventricular WMH extending into the deep white matter (Fazekas score 3) or confluent deep WMH (Fazekas score 2 or 3). EPVS number was counted in the dominant side of basal ganglia and graded into 4 levels (grade 0 for no EPVS, grade 1 for <10 EPVS, grade 2 for 11 to 20 EPVS, grade 3 for 21–40 EPVS, grade 4 for more than 40 EPVS) according to Semiquantitative scale, one point was awarded if there were moderate to severe (Semiquantitative grade 2–4) EPVS. The number of deep CMBs in the cerebellum, brainstem, basal ganglia, and thalamus was involved in this study, one point was awarded if there was one or more CMBs. One point was awarded if the presence of any sLI. The severity of the total CSVD burden was graded into three levels: mild (0 or 1 point), moderate (2 points) and severe (3 or 4 points) (Arba et al., 2017a; Liu et al., 2019). Cerebral atrophy was assessed using the Global Cortical Atrophy Scale (GCA), Moderate to severe brain atrophy was recorded as positive (2 or 3 points) (Pasquier et al., 1996).

### Statistical analysis

Continuous variables were compared by the Mann-Whitney *U* test for non-normally distributed variables and Student's *t*-test for normally distributed variables. Differences between proportions were assessed by Fisher's exact test or Chi-square test. Continuous variables were reported as the mean ± SD or median (interquartile range), and categorical variables were described by constituent ratio. The SPSS 20.0 was used for statistical analysis of baseline data. A univariable analysis was used to compare the baseline and clinical differences between the PSD and non-PSD groups. Variables with a *P* < 0.05 in univariable analysis were included in the multivariable logistic regression analysis. The nomogram is constructed by R version 4.0. Variables with a *P* < 0.05 in the multivariable logistic regression were entered into the nomogram. The nomogram was created by assigning a graphic preliminary score to each of the predictors with a point ranging from 0 to 100, which was then summed to generate a total score, finally converted to the logit, and then to an individual probability (from 0 to 100%) of early-onset PSD. The area under the receiver operating characteristic curve (AUC-ROC) was used to evaluate the differentiation of the prediction model. Calibration was tested using a calibration curve with bootstraps of 1,000 resamples, which reflected the agreement between nomogram and actual observation.

## Results

### Clinical features and baseline characteristics

A total of 346 patients were enrolled in the study, including 280 patients (80.9%) in the non-PSD group and 66 patients (19.1%) in the PSD group. The baseline characteristics of the patients in the two groups are shown in Table 1. In the non-PSD group, the median age was 63 (53–70) years old, and 67.5% were male. The median age was 64 (59–68) years old in the PSD group, with 53% being male. Univariate analysis showed that the differences between the two cohorts were gender, plasma fibrinogen, previous history of stroke, baseline NIHSS score, baseline BI, moderate to severe cerebral atrophy, and cognitive disorder (all *P* < 0.05).

**Table 1:** Baseline characteristics of patients in the non-PSD and PSD groups.

**Subjects**	**Non-PSD group** **(*n* = 280)**	**PSD group** **(*n* = 66)**	**Test value**	***P* value**
Age	63 (53–70)	64 (59–68)	−0.539	0.590
Gender, male (%)	189 (67.5)	35 (53)	4.899	0.027*
Baseline SBP, mmHg	146.52 ± 20.25	143.03 ± 20.60	1.256	0.210
Baseline DBP, mmHg	80 (72.5–90)	79 (74–87)	−0.391	0.695
**Laboratory exam**				
White blood cell*10^9^/L	6.83 (5.62–8.27)	7.19 (5.80–8.46)	−1.349	0.177
Neutrophil*10^9^/L	4.30 (3.31–5.66)	4.58 (3.63–5.38)	−0.386	0.699
Lymphocytes*10^9^/L	1.71 ± 0.60	1.88 ± 0.71	−1.924	0.055
Platelets*10^9^/L	195.42 ± 58.97	209.88 ± 64.29	−1.761	0.079
Total cholesterol mmol/l	4.33 ± 1.06	4.35 ± 0.88	−0.119	0.906
Triglyceride mmol/l	1.34 (1.02–1.88)	1.44 (1.00–1.95)	−0.181	0.856
LDL-C mmol/l	2.54 ± 0.89	2.58 ± 0.77	−0.278	0.781
HDL-C mmol/l	1.01 (0.85–1.18)	0.96 (0.81–1.12)	−1.348	0.178
Blood glucose mmol/L	5.20 (4.67–6.17)	5.00 (4.58–5.94)	−1.214	0.225
Total bilirubin μmol/l	14.30 (11.10–19.25)	13.45 (9.40–18.80)	−1.285	0.199
Uric acid mmol/l	308.55 ± 84.96	287.72 ± 89.79	1.772	0.077
Urea μmol/l	4.60 (3.90–5.60)	4.55 (3.90–6.00)	−0.404	0.686
Prealbumin mg/l	237.22 ± 51.06	227.73 ± 49.99	1.363	0.174
Albumin mg/l	40.43 ± 3.22	39.64 ± 2.76	1.850	0.065
Homocysteine μmol/l	9.00 (7.00–12.60)	10.30 (7.90–17.50)	−1.756	0.079
C-reactive protein mg/l	1.73 (0.84–4.13)	2.19 (0.78–5.32)	−0.704	0.482
Plasma fibrinogen g/l	2.58 ± 0.95	2.98 ± 0.88	−3.054	0.002*
**TOAST subtype cases (%)**			8.760	0.067
LAA	106 (37.9)	35 (53)		
CE	26 (9.3)	5 (7.6)		
SAO	92 (32.9)	20 (30.3)		
SOE	10 (3.6)	3 (4.5)		
SUE	46 (16.4)	3 (4.5)		
**Previous history cases (%)**				
Stroke	30 (10.7)	14 (21.2)	5.303	0.021*
Hypertension	196 (70.0)	45 (68.2)	0.084	0.773
Diabetes	64 (22.9)	16 (24.2)	0.058	0.810
Hyperlipidemia	36 (12.9)	5 (7.6)	1.426	0.232
Atrial fibrillation	29 (10.4)	4 (6.1)	1.143	0.285
Coronary heart disease	6 (2.1)	0 (0.0)	1.439	0.230
Smoking	78 (27.9)	12 (18.2)	2.598	0.107
Alcohol abuse	53 (18.9)	9 (13.6)	1.017	0.313
Base NIHSS score	2 (1–4)	3 (1–6)	−2.211	0.027*
Baseline BI	95 (65–95)	65 (50–95)	−2.801	0.005*
**Degree of education (%)**			1.680	0.432
Illiteracy	21 (7.5)	8 (12.1)		
Primary school	157 (56.1)	37 (56.1)		
Middle school and higher	102 (36.4)	21 (31.8)		
Cognitive disorder cases (%)	43 (15.4)	24 (36.4)	15.094	<0.001*
**Position of the focal (%)**				
Left hemisphere	122 (43.6)	32 (48.5)	0.522	0.470
Left frontal lobe	35 (12.5)	12 (18.2)	1.469	0.226
Left basal ganglia	48 (17.1)	11 (16.7)	0.009	0.926
Frontal lobe	65 (23.2)	18 (21.7)	0.482	0.487
Parietal lobe	66 (23.6)	17 (25.8)	0.140	0.708
Temporal lobe	53 (18.9)	18 (27.3)	2.280	0.131
Occipital lobe	51 (18.2)	11 (16.7)	0.087	0.768
Basal ganglia	93 (33.2)	23 (34.8)	0.064	0.800
Brain stem	56 (20.0)	13 (19.7)	0.003	0.956
Thalamus	33 (11.8)	5 (7.6)	0.968	0.325
Cerebellum	32 (11.4)	6 (9.1)	0.299	0.585
Moderate to severe cerebral atrophy (%)	32 (11.4)	17 (25.8)	9.021	0.003*

SBP systolic blood pressure, DBP diastolic blood pressure, LDL-C low-density lipoprotein, HDL-C high-density lipoprotein, NIHSS National Institutes of Health Stroke Scale, BI Barthel Index, LAA large artery atherosclerosis, CE cardio embolism, SAO small artery occlusion, SOE stroke of other demonstrated etiology, SUE stroke of other undetermined etiology.

Continuous data were shown as mean ± SD or median (interquartile range). Categorical variables were described by constituent ratio.

* *P* < 0.05.

### The relationship between early-onset PSD and CSVD

**Figure 1:**
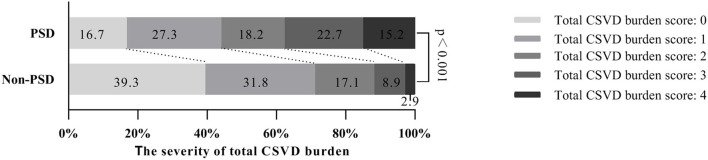
Total CSVD burden score distribution between the two cohorts. CSVD, cerebral small vessel disease.

In our study, moderate to severe WMHs were found in 36.1% of the subjects, sLI were in 17.1% of the subjects, CMBs were in 27.5% of the subjects and EPVS grades 2–4 were in 40.5%. In the PSD group, total CSVD burden assessment showed that 27.3% were 1 score, 18.2% were 2 score, 22.7% were 3 and 15.2% were 4, while in the non-PSD group, 31.8% were 1 score, 17.1% were 2, 8.9% were 3 and 2.9% were 4 (Figure 1). Univariate analysis showed that mild to severe WMHs, EPVS grades 2–4, the existence of sLI or CMBs, and total CSVD burden score were different between the non-PSD and PSD groups (Table 2).

**Table 2:** The relationship between early-onset PSD and CSVD.

**Imaging manifestations**	**Non-PSD group** **(*n* = 280)**	**PSD group** **(*n* = 66)**	**Test value**	***P* value**
**Moderate to severe WMHs**	36 (12.9)	23 (34.8)	18.262	<0.001*
**sLI**	93 (33.2)	32 (48.5)	5.397	0.020*
**EPVS grade 2–4**	97 (34.6)	43 (65.2)	20.636	<0.001*
**CMBs**	66 (23.6)	29 (43.9)	11.124	0.001*
**CSVD total burden score**			32.514	<0.001*
0 point	110 (39.3)	11 (16.7)		
1 point	89 (31.8)	18 (27.3)		
2 points	48 (17.1)	12 (18.2)		
3 points	25 (8.9)	15 (22.7)		
4 points	8 (2.9)	10 (15.2)		

WMHs, white matter hyperintensities; CMBs, cerebral microbleeds; EPVS, enlarged perivascular spaces; sLI, silent lacunar infarction; CSVD, cerebral small vessel disease.

* *P* < 0.05.

### The independent risk factors of early-onset PSD

Variables with a *P* < 0.05 were included in the multivariable logistic regression model. The results indicate that gender (OR = 0.440, 95%CI: 0.238–0.813), cognitive disorder (OR = 2.501, 95%CI: 1.283–4.876), baseline BI (OR = 0.986, 95%CI: 0.975–0.997), plasma fibrinogen concentration (OR = 1.425, 95%CI: 1.050–1.933) were independently related to early-onset PSD. In reference to the 0 score of the total CSVD burden, 2–4 points (moderate to severe total CSVD burden) was an independent risk factor of early-onset PSD and the higher the point was, the higher the OR value was, which meant a higher risk of the occurrence of early-onset PSD (Table 3).

**Table 3:** Multivariable logistic regression of early-onset PSD.

**Subjects**	**Full multivariable model OR (95%CI)**	***P* value**
**Gender, male**	0.440 (0.238–0.813)	0.009*
**Cognitive disorder**	2.501 (1.283–4.876)	0.007*
**Previous stroke**	1.275 (0.549–2.960)	0.573
**Baseline NIHSS score**	0.937 (0.818–1.074)	0.350
**Baseline BI**	0.986 (0.975–0.997)	0.012*
**Moderate to severe cerebral atrophy**	1.405 (0.638–3.092)	0.398
**Plasma fibrinogen**	1.425 (1.050–1.933)	0.023*
**Total CSVD burden score**		<0.001*
0 point	Ref.	Ref.
1 point	2.146 (0.925–4.976)	0.075
2 points	2.632 (1.028–6.735)	0.044*
3 points	5.638 (2.171–14.643)	<0.001*
4 points	12.851 (3.920–42.129)	<0.001*

NIHSS, National Institutes of Health Stroke Scale; BI, Barthel Index; CSVD, cerebral small vessel disease.

* *P* < 0.05.

### Prediction nomogram for early-onset PSD

**Figure 2:**
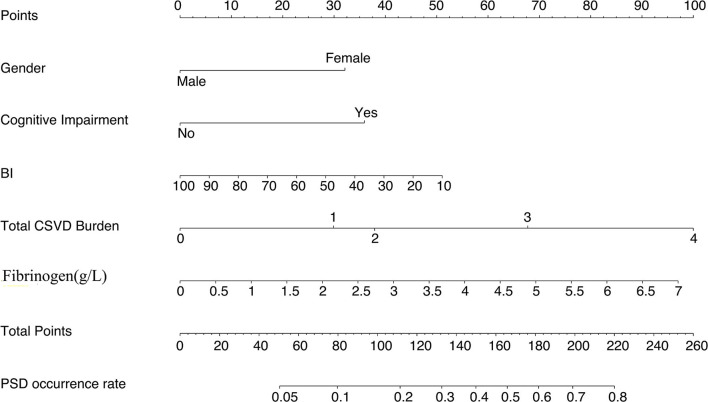
Nomogram predicting early-onset PSD. BI, Barthel Index.

The nomogram consisted of the variables that had *P* < 0.05 in the multivariable logistic regression (Figure 2). It was generated by assigning a graphic preliminary score to each of the 5 factors (gender, cognitive disorder, baseline BI, fibrinogen, and moderate to severe total CSVD burden score) with a point ranging from 0 to 100. Then all the scores were summed up to generate a total score and finally converted into an individual probability of the onset of early-onset PSD. The probability was from 0 to 100%.

### The discrimination and calibration performance of the model

**Figure 3:**
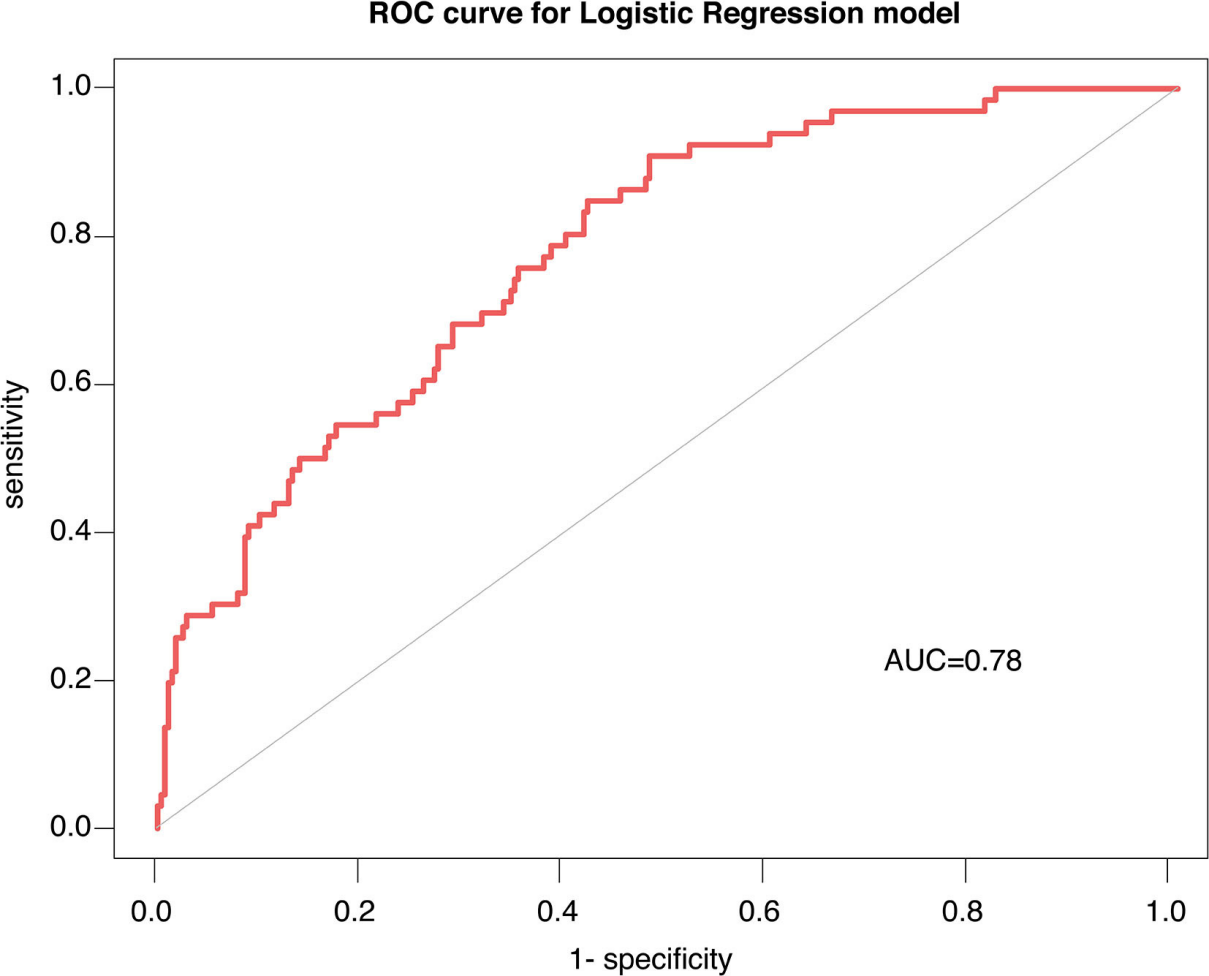
Receiver operating characteristic curve (ROC) analysis for the nomogram.

**Figure 4:**
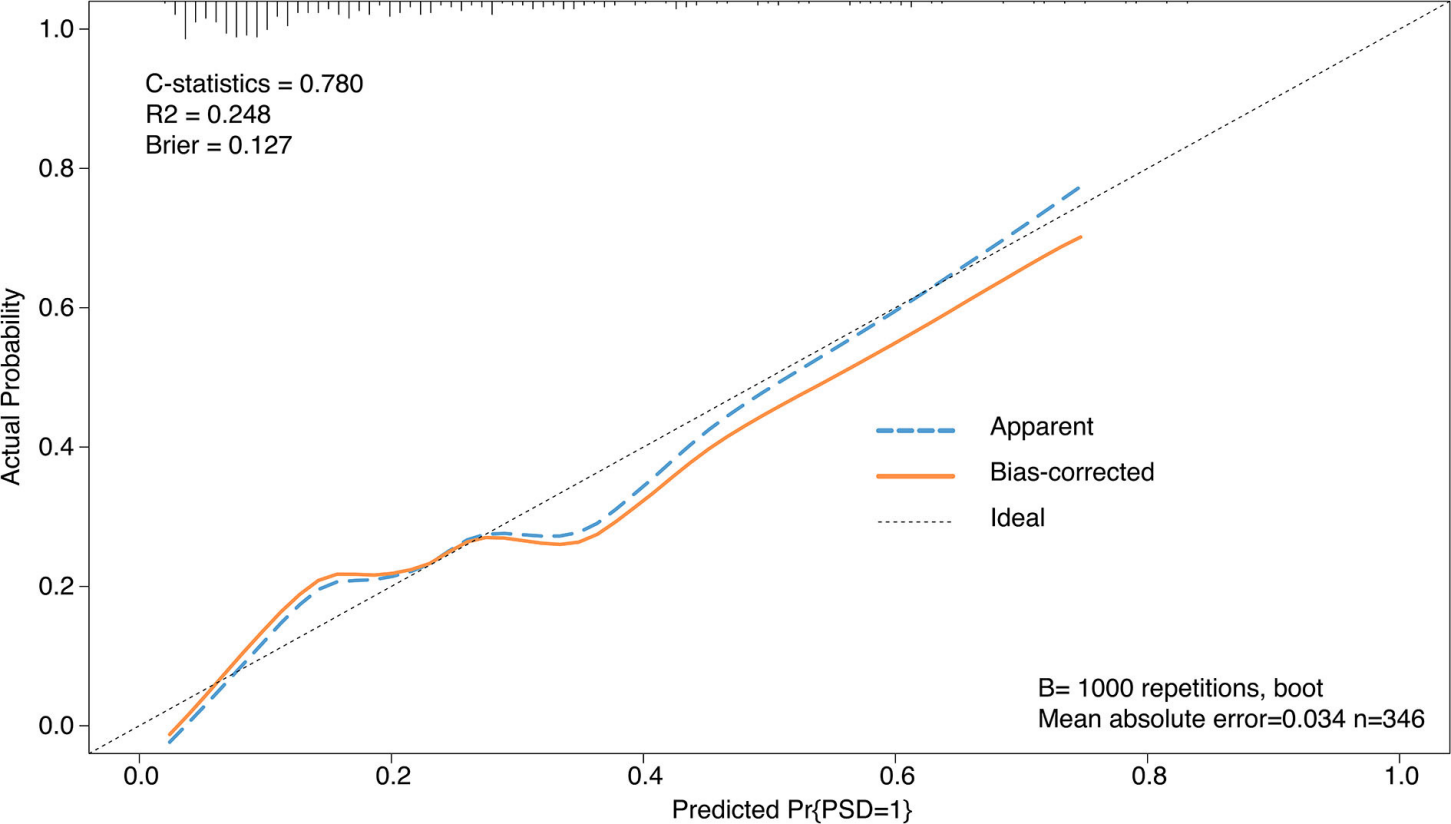
The calibration curves for the nomogram.

The C-Statistics was used to assess the discriminative capacity of the nomogram as demonstrated in Figure 3. The AUC was 0.780 (95%CI 0.751–0.810). The calibration curves were demonstrated in the Figure 4. The x-axis exhibited the predicted possibility of PSD, and the y-axis represented the actual possibility of PSD. The calibration plot revealed a general fit of the nomogram predicting the risk of early-onset depression in patients with AIS.

## Discussion

PSD is a specific type of depression that can be classified into depressive disorders caused by other physical disorders according to the DSM-5 (Mittal and Walker, 2011). In 1975, a study conducted by Robinson et al. indicated that decreased cerebral blood flow could affect catecholamine concentration and induce “fight or flight” phenomenon (Robinson et al., 1975). Folstein et al. (1977) found that about 1/2 of the stroke patients suffered from depression, while only 1/5 of orthopedic patients suffered from depression to the same disability degree. These findings inspire neurologists to explore the relationship between depression and cerebral injury.

Early-onset PSD refers to the depression that occurs within 2 weeks after the onset of stroke. Compared to late-onset PSD, early-onset PSD possesses more depression symptoms and may affect patients' prognosis (Tateno et al., 2002; Willey et al., 2010). Large amounts of guidelines and expert consensus pointed out the importance of early diagnosis and treatment of PSD. For instance, the European Guidelines for stroke management, published in 2008 emphasizes that doctors must pay more attention to the early diagnosis of PSD (ESO, 2008). Canadian Guidelines for the best management of stroke delivered in 2009 also point out that depression screening is recommended before discharge for patients in the acute phase of stroke (Lanctôt et al., 2020).

Our study indicated that the four imaging subtypes of CSVD, including WMHs, sLI, CMBs, and EPVS, were all related to the early-onset PSD. Current studies also indicate that the CSVD subtypes are related to PSD. WMHs were the most representative imaging marker of CSVD. A case-control study conducted by Tang et al. illuminated that WMHs were related to PSD 3 months after a stroke attack (Tang et al., 2010). Another study that followed up 294 lacunar infarction patients with CSVD found that the severity of baseline WMHs was independently related to late-onset PSD (Pavlovic et al., 2016). An up-to-date research also concluded that deep WMHs were an independent risk factor of PSD in TIA or mild stroke patients (Carnes-Vendrell et al., 2019). sLI is also called asymptomatic lacunar infarcts or lacunes. Santos et al. revealed that the accumulative chronic lacunes in the thalamus, basal ganglia, and deep white matter were obviously related to PSD onset rather than single infarction by conducting an autopsy (Santos et al., 2009a). Another study on patients with acute lacunar infarction found that baseline sLI was independently related to PSD 3 months after stroke onset (Zhang et al., 2017). The study of the relationship between deep CMBs and PSD is rare. Tang et al. reported that lobular CMBs were the independent risk factor of PSD 3 months after stroke onset (Tang et al., 2014) and the number of lobular CMBs was positively related to PSD severity (Tang et al., 2011). The relationship between basal ganglia EPVS and PSD is not clear yet. Zhang et al. indicated that severe EPVS was not independently related to PSD (Zhang et al., 2017). Another 3-months prospective study targeting AIS patients indicated that severe EPVS in the basal ganglia region was not related to PSD, while centrum semiovale EPVS was remarkably related to PSD (Liang et al., 2018a). To sum up, the relationship between the imaging manifestations of CSVD subtypes and PSD is not better researched and heterogenicity is prominent, thus, further research is required in this field.

In our study, about 1/3 of patients possessed 2 or more characteristics, which was similar to the distribution of CSVD overall burden in other studies. As mentioned before, different CSVD imaging characteristics usually coexist in one patient, and CSVD overall scale is more clinically significant than a single CSVD type. Hence, a rating system that involves multiple CSVD types can better evaluate the overall burden of CSVD (Staals et al., 2014). However, so far, only a few studies have explored the relationship between total CSVD burden and PSD. Zhang et al. found that CSVD overall burden was an independent prediction factor of PSD while the research only focused on patients that first suffered from lacunar infarction (Zhang et al., 2017). Another study explored the relationship between baseline total CSVD burden and poststroke depressive symptoms (PDS) (Liang et al., 2018b). The study involved 563 mild AIS patients and followed them on the 3th, 9th and 15th month. The results showed that the baseline total CSVD burden was positively related to PSD risk at all these three periods of the follow-up visit. Another study reported that CSVD burden not only directly determined PSD symptoms but also worsened stroke severity, and post-stroke neurological deficits (Liang et al., 2019). To our knowledge, there has not been any research that focuses on the relationship between total CSVD burden and early-onset PSD. In this research, we found that 2–4 points of total CSVD burden were obviously related to early-onset PSD and the patients with higher total CSVD burden were more likely to suffer from early-onset PSD.

Several mechanisms could explain the relationship between CSVD and PSD. First of all, patients with pre-stroke CSVD often suffer from more vascular risk factors such as hypertension, diabetes, and so on, which are proven to be closely related to brain dysfunction (Muresanu et al., 2014). Besides, CSVD means the chronic cumulative ischemia of the brain, which may cause damage to hemodynamic neurovascular coupling (Monteiro et al., 2021; Yang et al., 2022). The hemodynamic neurovascular coupling ensures a strong increase in cerebral blood flow and a fast increase in neuronal glucose uptake in the case of acute incidents of the brain (Popa-Wagner et al., 2015). When patients with CSVD suffer from a sudden attack of acute ischemic stroke, the compensatory ability of the brain is weaker, thus, it is more likely to damage the depression-related network such as the frontal-subcortical circuit (Brookes et al., 2014; Arba et al., 2017b; van Agtmaal et al., 2017; Gu et al., 2022). In addition, local vascular inflammation contributes to the development of PSD (Spalletta et al., 2006). Pre-stroke CSVD may worsen the immune-inflammatory activation following AIS (Licata et al., 2006), which promotes depression.

There were several studies that built nomograms to predict PSD. Lan et al. identified that HAMD-17 scores, APTT, serum direct bilirubin, and FT4 at admission were related to the onset of persistent PSD in the first year and developed a nomogram to predict the risk of persistent PSD, which was proved to have good clinical reliability (Lan et al., 2022). Li et al. developed and validated a nomogram to predict the risk of major post-stroke depression at 3 months after AIS onset, which could be used to promote the early screening of PSD and was clinically practical (Li et al., 2021). Another study constructed an effective nomogram to predict the probability of PSD based on magnetic resonance spectroscopy (Qiao et al., 2020). To our best knowledge, this study was the first attempt to establish a practical and visual prediction model of early-onset PSD. The nomogram prediction model based on total CSVD burden score, gender, cognitive disorder, baseline BI and fibrinogen concentration had a C statistical magnitude of 0.780 and could predict early-onset PSD onset well. Thus, our nomogram model could increase early-onset PSD diagnosis efficiency and help doctors develop management and prevention strategies for PSD.

However, there were still deficiencies in our study. First, the study was a monocentric and small sample research. Bias might exist due to a lack of samples. In addition, since it was difficult to distinguish whether depression symptoms were caused by stroke or previous depression, patients with a depression medical history were not included in the research, which might have influenced our results. Besides, several patients were excluded from our study for the reason of aphasia or disturbance of consciousness or dementia at admission, and yet these patients may have experienced more severe strokes. Thus, patients included in our study may have had a relatively mild stroke attack. This might explain why there was no significant association between NIHSS score and early-onset PSD in our study to some extent. Finally, the nomogram designed by the research was barely applied to the prediction of early-onset PSD in the acute phase. Whether the nomogram could be used to predict PSD risk in different phases was not verified. Therefore, we would conduct careful follow-ups on patients involved in further research.

## Conclusion

We found that a total CSVD burden score of 2–4 points was an independent risk factor of early-onset PSD. We also proposed and validated a nomogram model that included total CSVD burden score, gender, cognition disorder, baseline BI and plasma fibrinogen concentration, which reliably assessed the probability of early-onset depression in AIS patients.

## Data availability statement

The original contributions presented in the study are included in the article/supplementary material, further inquiries can be directed to the corresponding authors.

## Ethics statement

The studies involving human participants were reviewed and approved by the Ethics Committee of the First Hospital Affiliated to Soochow University. Written informed consent for participation was not required for this study in accordance with the national legislation and the institutional requirements.

## Author contributions

TL and QF conceived and designed the research. LZ and LC analyzed the data and drafted the manuscript. SD and YQ collected the data and performed the research. LM collected and analyzed the data. All authors reviewed and edited the manuscript and approved the final version of the manuscript.

## Funding

This study was supported by the National Natural Science Foundation of China (82071300), the Suzhou Science and Technology Development Plan (SYSD2020073), and the Stroke Team of Professor QF from the First Affiliated Hospital of Soochow University (SZYQTD202106).

## Conflict of interest

The authors declare that the research was conducted in the absence of any commercial or financial relationships that could be construed as a potential conflict of interest.

## Publisher's note

All claims expressed in this article are solely those of the authors and do not necessarily represent those of their affiliated organizations, or those of the publisher, the editors and the reviewers. Any product that may be evaluated in this article, or claim that may be made by its manufacturer, is not guaranteed or endorsed by the publisher.

## Abbreviations

AIS: acute ischemic stroke
AUC-ROC: area under the receiver operating characteristic curve
BI: Barthel index
CMBs: cerebral microbleeds
CSVD: cerebral small vessel disease
DSM-5: Diagnostic and Statistical Manual of Mental Disorders Version 5
DWI: diffusion-weighted imaging
EPVS: enlarged perivascular spaces
HAMD-17: 17-item Hamilton Depression scale
MMSE: Mini-Mental State Examination
MRI: magnetic resonance imaging
PDS: poststroke depressive symptoms
PSD: post-stroke depression
sLI: silent lacunar infarction
SWI: susceptibility weighted imaging
WMHs: white matter hyperintensities.
